# Comparison of side-chain dispersion in protein structures determined by cryo-EM and X-ray crystallography

**DOI:** 10.1107/S2052252521011945

**Published:** 2021-12-10

**Authors:** Ashraya Ravikumar, Mrugsen Nagsen Gopnarayan, Sriram Subramaniam, Narayanaswamy Srinivasan

**Affiliations:** a Molecular Biophysics Unit, Indian Institute of Science, Bengaluru, India; b Undergraduate studies, Indian Institute of Science, Bengaluru, India; c University of British Columbia, Vancouver, British Columbia V6T 1Z3, Canada

**Keywords:** single-particle cryo-EM, structure determination, cryo-electron microscopy, protein structure, X-ray crystallography

## Abstract

An evaluation of systematic differences in local structure and conformation in the interior of protein tertiary structures determined by crystallography and by cryo-electron microscopy (cryo-EM) is reported. The expectation is that any consistent differences between the derived atomic models could provide insights into variations in side-chain packing that result from differences in specimens prepared for analysis between these two methods. By computing an atomic packing score, which provides a quantitative measure of clustering of side-chain atoms in the core of the tertiary structures, it is found that, in general, for structures determined by cryo-EM, side chains are more dispersed than in structures determined by X-ray crystallography over a similar resolution range. This trend is also observed in the packing comparison at subunit interfaces. Similar trends were observed in the packing comparison at the core of tertiary structures of the same proteins determined by both X-ray and cryo-EM methods. It is proposed here that the reduced dispersion of side chains in protein crystals could be due to some level of dehydration in 3D crystals prepared for X-ray crystallography and also because the higher rate of freezing of protein samples for cryo-EM may enable preservation of a more native conformation.

## Abstract 2

A comparison of atomic packing scores in protein structures determined using cryo-electron microscopy (cryo-EM) and X-ray crystallography reveals that side chains are more dispersed, indicated by lower packing scores, in cryo-EM structures compared with crystal structures.

## 1. Introduction

The 3D structures of proteins play a major role in understanding their functional, regulatory and mechanistic features. To that end, X-ray crystallography has contributed richly for several decades to further our knowledge on protein structures, with nuclear magnetic resonance (NMR) being a later and important addition. Over the past few years, cryo-electron microscopy (cryo-EM) has advanced as yet another successful experimental method to determine 3D structures, especially of large macromolecular assemblies (Egelman, 2016 ▸; Kühlbrandt, 2014 ▸). Recent advances in technology and method­ology involved in single particle cryo-EM (Li *et al.*, 2013 ▸; Faruqi *et al.*, 2003 ▸) have enabled structure determination of smaller proteins and large assemblies at near-atomic resolution (Merk *et al.*, 2016 ▸, 2020 ▸; Wu *et al.*, 2020 ▸; Hamaguchi *et al.*, 2019 ▸).

Although the end goal for protein X-ray crystallography, NMR and cryo-EM is to determine the 3D structures of proteins or assemblies of proteins, the methods themselves are quite different from one another, starting from the sample preparation stage to the structure refinement stage. For instance, in the sample preparation stage for NMR, the protein structure is determined in aqueous solution at a temperature above 0°C; in cryo-EM, the protein sample in aqueous solution is placed on a grid and flash-frozen before data collection by electron microscopy; in X-ray crystallography, a super-saturated solution of the protein is coaxed to crystallize and then flash-frozen before bombardment with X-rays for data collection. Each of these sample-preparation techniques could have its own effect on the conformation and dynamical features of proteins.

In this work, we assess the differences, if any, in local structure and conformation in the interior (core of the tertiary structures) of the proteins studied by X-ray crystallography and cryo-EM, with the expectation that the differences, if any, will provide an insight into protein structural changes that result from the way specimens are prepared for analysis by these two methods. Protein structures determined by the different modalities are influenced by the specific method­ology and the physical state of the protein under which the measurement is carried out. A method-independent parameter to study such effects is the extent of local compaction or dispersion of side chains in the 3D structure of proteins, as indicated by the packing of atoms in the proteins. We measure this local clustering of buried atoms in the tertiary structure, which we term ‘atomic packing score’, using the Voronoi cell method (Richards, 1974 ▸). This is a measure of local dispersion of the side chains because it is essentially a calculation of the density of neighboring atoms at each atomic position. Thus, given two volumes with identical numbers of atoms, the atomic packing score will be higher if these atoms are locally clustered compared with a situation where they are evenly dispersed in the same volume. We analyze the atomic packing scores of cryo-EM structures and compare them with those of crystal structures, both at the tertiary structural level and at protein–protein interfaces. We calculated the packing score only for the buried atoms either in the tertiary structure or in the protein–protein interface. We also avoid biases due to resolution by comparing structures solved in the same resolution range and biases attributed to difference in size and nature of protein by performing pairwise packing score comparisons of the same protein solved by both cryo-EM and X-ray crystallography.

## 2. Materials and methods

### 2.1. Dataset of cryo-EM and crystal structures

We considered all protein and protein assembly structures determined by cryo-EM with reported resolutions ≤3.5 Å with atomic level models fitted in the density maps and deposited in the Protein Data Bank (PDB) (Berman *et al.*, 2000 ▸) as of May 2019. From these, we filtered out highly symmetric structures such as viral capsids that could bias the dataset for analysis on atomic packing score. This resulted in 317 cryo-EM structures for the current analysis. Previous studies on the packing of crystal structures show a strong correlation between packing score and resolution of the structures (Seeliger & de Groot, 2007 ▸). Hence we created a dataset of crystal structures of protein complexes with crystallographic resolutions >2 Å and ≤3.5 Å, which is the resolution range of the cryo-EM structures dataset. We also filtered out structures with chain lengths less than 30 residues. As the number of crystal structures satisfying these criteria is huge (>14 000), we randomly chose roughly the same number of crystal structures (300) as the number of cryo-EM structures used in our analysis. We did this random selection three times to ensure robustness of the results. Therefore, the number of crystal structures and their resolution range is comparable to the dataset of cryo-EM structures. These three random sets of crystal structures are referred to as Set 1, Set 2 and Set 3 throughout the paper. The lists of cryo-EM structures and crystal structures used in the analysis are given in Tables S1 and S2 of the supporting information, respectively.

### 2.2. Identification of interfaces between interacting proteins in the complexes

From the dataset of cryo-EM structures, the sequences of each chain in the structures were clustered at 100% sequence identity using *CD-HIT* (Huang *et al.*, 2010 ▸), and non-identical pairs of chains in each structure were identified. Core interface residues were identified from these pairs based on the following criteria: a residue with relative solvent accessibility ≤7% in the complexed form and ≥10% in the ‘uncomplexed’ form is considered an interface residue (De *et al.*, 2005 ▸). Solvent accessibility was calculated using *NACCESS* (Hubbard & Thornton, 1993 ▸). Among these interfaces, we filtered out interfaces with missing regions close to the interface using the following criteria: if there was a missing residue within three residues preceding or succeeding an interface residue, then that interface was discarded from further analysis. Then, we identified those interface residues which are involved in short contacts with another residue in the structure using the clashscore module in *MolProbity* (Davis *et al.*, 2007 ▸). Two non-bonded atoms are said to be clashing if their overlap is ≥0.4 Å. These residues were not considered for calculation of the average atomic packing score of the interfaces since they could result in an artificially high packing score. Finally, only those interfaces in which each chain of a pair contributed at least two residues to the interface were retained for analysis. All the above criteria and filters were also applied to crystal structures. In total, 946 interfaces from cryo-EM structures and an average of 787 interfaces (601 in Set 1, 750 in Set 2 and 1009 in Set 3) from crystal structures were analyzed.

### 2.3. Atomic packing score calculation

We used *Voronoia* (Rother *et al.*, 2009 ▸) with a grid distance of 0.1 Å to calculate the atomic packing score (also known as packing density in the literature) of the cryo-EM and crystal structures. Only protein atoms and atoms with ‘A’ occupancy in the case of multiple occupancy were considered for the packing score calculation. The packing score for every buried residue was obtained by averaging the packing score of constituent atoms of the residue. Buried atoms in the protein were identified by the *Voronoia* tool, by considering those atoms which cannot make contact with a 1.4 Å radius water probe rolled along the surface of the protein. To account for the effect of missing side-chain atoms on the packing scores, we modeled such atoms using *Modeler* (Webb & Sali, 2016 ▸) and then compared the packing scores of every residue in the completed structure and original structure. If there was a difference in packing of the residue at the first decimal position, then that residue was discarded from the average packing score calculation. Otherwise, the packing score of the residue from the original structure was used for the average score calculation. We also filtered out residues identified as rotamer outliers by *MolProbity* from the average packing score calculation (Hintze *et al.*, 2016 ▸).

## 3. Results and discussion

### 3.1. Overall atomic packing in cryo-EM structures

**Figure 1:**
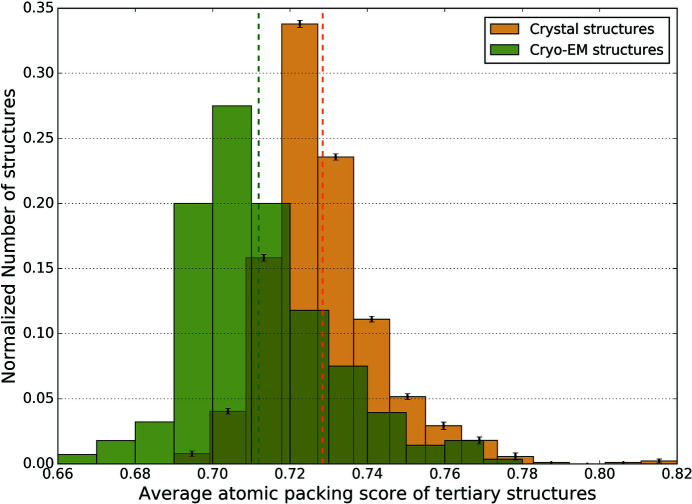
Average atomic packing scores for cryo-EM and crystal structures. The distribution of the average atomic packing scores of buried residues in the tertiary structures is shown for cryo-EM (green) and X-ray (yellow) structures. The *y* axis values are obtained by normalizing the number of structures in each bin of the histogram with respect to the total number of structures. The mean values of the two distributions are shown by the dotted lines in their respective colors. The error bars on the histogram of the crystal structures indicate the standard deviation of the number of structures in the corresponding bins across the three sets of randomly chosen crystal structures.

A commonly used method to study atomic packing is the Voronoi cell method, which defines the solvent-excluded volume and van der Waals volume of each atom (Richards, 1974 ▸; Gerstein *et al.*, 1995 ▸; Goede *et al.*, 1997 ▸). The packing score of an atom is the ratio between the van der Waals volume and the sum of the van der Waals volume and the solvent-excluded volume. Though this ratio is commonly termed packing density, it is essentially a measure of the spacing of atoms within the protein. Hence in this paper, we refer to it as an atomic packing score. We calculated atomic packing scores for every atom buried in the tertiary structures of cryo-EM and crystal entries in our datasets and obtained an average atomic packing score for each structure, after filtering out residues whose packing is affected by missing atoms in the neighborhood and residues with side-chain rotamer outliers. Fig. 1 ▸ shows a histogram of the average atomic packing score of buried residues in cryo-EM structures, along with crystal structures. The error bars in the histogram of crystal structures denote the standard deviation in the number of structures in each bin across the three sets of randomly chosen crystal structures (the histograms of the three sets are shown separately in Fig. S1 of the supporting information). The distribution of scores for the cryo-EM structures is shifted to lower values compared with crystal structures, indicating that most cryo-EM structures are more loosely packed in the interior than crystal structures.

To further avoid the effect of incorrectly modeled residues in packing scores, we also used *WHATCHECK* to identify residues that are in an unusual environment, determined based on nature of neighboring residues (Vriend & Sander, 1993 ▸). We filtered out such residues and calculated the average packing of tertiary structures. Here too, we find that cryo-EM structures (average packing score = 0.712) are more loosely packed than crystal structures (average packing score = 0.727). This comparison is shown in Fig. S2.

The mean atomic packing score of all cryo-EM structures is 0.712, whereas for crystal structures it is 0.729 and the difference in distribution is statistically significant (*p* < 10^−16^; Mann–Whitney Test). There is also some overlap between the two distributions. Hence, though cryo-EM structures generally have lower packing than crystal structures, there could be some instances of cryo-EM structures where the average packing score is comparable to that of crystal structures.

Although the resolution range of the cryo-EM and crystal structure dataset used in this study is the same (∼2 to 3.5 Å), we further categorized the structures into three resolution ranges: <2.5 Å, between 2.5 and 3 Å, and ≥ 3 Å. On comparing the packing scores of tertiary structures of crystal and cryo-EM structures in these sub-categories, we once again find that side chains in cryo-EM structures are significantly more dispersed and less tightly packed than crystal structures [*p* < 0.05, Mann–Whitney Test (exact values shown in Table 1 ▸)]. The histograms of packing scores in these three sub-categories of resolution and the three sets of crystal structures compared with the cryo-EM structures are shown in Fig. S3. We also compared packing scores in finer resolution bins (0.2 Å width) and note the same trend as seen in Table 1 ▸. These comparisons are shown in Table S3.

**Figure 2:**
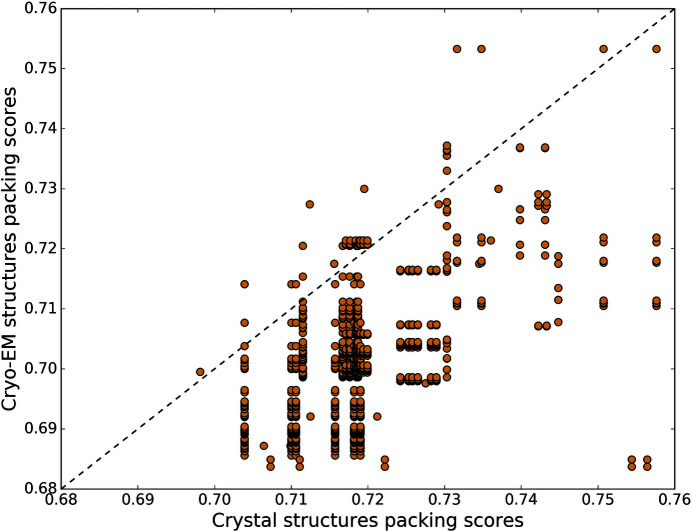
Packing scores of pairs of the same proteins solved by cryo-EM and X-ray crystallography. Points below the diagonal line (packing of the cryo-EM structure equal to the crystal structure) are cases where the crystal structure (in Set 1) has a higher packing score compared with its cryo-EM counterpart. In total, 92% of the points are below the line. Plots for the remaining sets are shown in Fig. S4.

An overall comparison of packing scores between cryo-EM and crystal structures could be biased by the different nature and size of proteins in the dataset. Hence, we identified pairs of cryo-EM and crystal structures of the same protein (at the tertiary structure level) and compared their packing scores. We note that in more than 90% of such pairs, the crystal structure has a higher packing score compared with the cryo-EM structure. Fig. 2 ▸ shows the packing scores of pairs of cryo-EM and crystal structures (Set 1). In total 92% of points fall below the *y* = *x* line, indicating that, in all these cases, the cryo-EM structure has a lower average packing score than the crystal structure. The plots for sets 2 and 3 are shown in Fig. S4, where cryo-EM structures have lower packing scores in 89% and 98% of pairs, respectively. To further account for any bias in this comparison due to difference in resolution between pairs of structures, we compared the packing scores of those pairs whose difference in resolution is <0.5 Å. We see that, in all three sets, an overwhelming majority of crystal structures are more tightly packed than their cryo-EM counterparts (Table S4). We also exclusively compared the packing scores of membrane protein structures solved by X-ray crystallography as well as cryo-EM. In 95% of the membrane proteins, the crystal structure has higher a packing score compared with their cryo-EM counterparts.

Several previous studies have examined the effects of crystallization and cooling of crystals to very low temperatures on the structure of protein trapped within the crystals (Earnest *et al.*, 1991 ▸; Frauenfelder *et al.*, 1987 ▸; Tilton *et al.*, 1992 ▸; Kurinov & Harrison, 1995 ▸; Juers & Matthews, 2001 ▸, 2004 ▸; Skrzypczak-Jankun *et al.*, 2006 ▸; Edayathumangalam & Luger, 2005 ▸; Fraser *et al.*, 2011 ▸). Protein crystals are obtained from super-saturated solutions of protein, and their crystals have 50% water content on average (McPherson & Gavira, 2014 ▸). Apart from reduced water content, cooling the crystals to very low temperature, close to the boiling point of nitro­gen, causes further dehydration accompanied by a reduction in unit-cell volume (Edayathumangalam & Luger, 2005 ▸), increases intermolecular contacts within crystals by making the side chains at the periphery of the molecule more ordered (Juers & Matthews, 2001 ▸, 2004 ▸; Bartesaghi *et al.*, 2014 ▸) and causes the molecule itself to contract (Frauenfelder *et al.*, 1987 ▸). The effects of crystal cooling are also seen within the protein where reduction in volume of internal cavities has been noted due to side chains of residues being brought closer together (Fraser *et al.*, 2011 ▸; Skrzypczak-Jankun *et al.*, 2006 ▸). Even though cryo-EM samples also undergo plunge-freezing before data collection, there are fundamental differences in characteristics of the protein sample. In cryo-EM samples, the proteins are in an aqueous medium just before freezing and compared with the slow freezing experienced by 3D crystals that are cooled at cryogenic temperatures, the speed at which the samples are cooled for cryo-EM analysis is much more rapid, with an estimated rate of temperature change of ∼10^6^ K s^−1^ (Dubochet *et al.*, 1988 ▸). This rapid rate is achieved using cryogens such as liquid ethane with boiling point temperatures that are ∼100° higher than nitro­gen, thereby enabling much more rapid cooling by vitrification that retains a hydrogen bonding environment comparable to that in an aqueous solution. The systematic and significant lower packing score observed for cryo-EM structures compared with crystal structures suggests that side chains in proteins are more dispersed and less closely packed under native conditions than previously thought based on crystal structures.

### 3.2. Atomic packing scores at protein–protein interaction interfaces

So far, we have presented an analysis of cryo-EM determined structures at the level of buried residues in the tertiary structures. However, almost all cryo-EM structures are from macromolecular assemblies consisting of several subunits/chains interacting with each other. It is also well known that for many cryo-EM structures, the final model is obtained by fitting existing crystal structures of these individual subunits into the potential maps or by modeling these subunits based on homologous template structures (Malhotra *et al.*, 2019 ▸). Hence there is a possibility of bias from existing crystal structures at the tertiary structural level. With this in mind, we analyzed interfaces between non-identical polypeptide chains in cryo-EM structures and crystal structures. All the interface residues considered for analysis are the well buried interacting residues, which are identified based on relative solvent accessibility in bound and unbound forms (see Materials and methods[Sec sec2] for further details).

**Figure 3:**
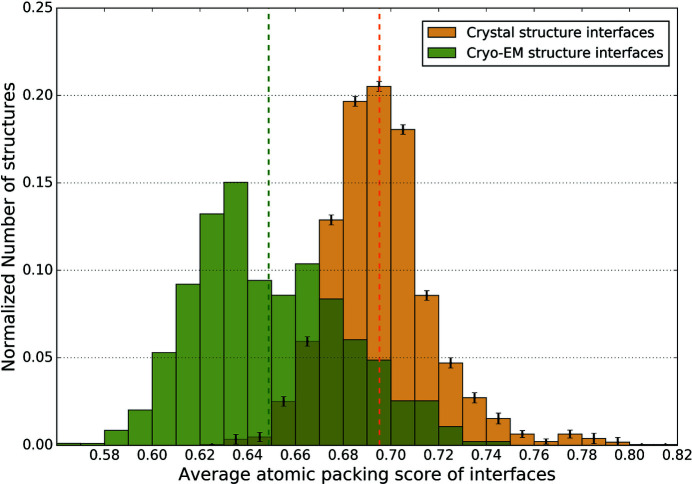
Average atomic packing score of interface residues in cryo-EM and crystal structures. This plot is similar to that in Fig. 1 ▸, except that it shows the distribution of the average atomic packing score of residues in interfaces between non-identical polypeptide chains in cryo-EM and crystal structures of assemblies.

It is known from previous work that packing densities at protein–protein interfaces are lower than within tertiary structures (Sonavane & Chakrabarti, 2008 ▸). In accordance with this, we observed that the mean atomic packing score of cryo-EM interfaces is lower than the tertiary structures (0.649 and 0.712, respectively). Importantly, we compared the atomic packing scores at the protein–protein interfaces obtained from cryo-EM and crystal structures. Fig. 3 ▸ shows the distribution of the average interface packing score for cryo-EM and crystal structure interfaces (the error bars in the histogram of crystal structures denote the standard deviation in the number of interfaces in each bin across the three sets of randomly chosen crystal structures). The histograms of the three sets are shown separately in Fig. S5. Note that the mean atomic packing score of interfaces in cryo-EM structures (0.649) is significantly lower than that of interfaces in crystal structures (0.695) (*p* < 10^−16^; Mann–Whitney Test). The distribution of interface packing scores of cryo-EM structures is shifted to lower values compared with crystal structures. Hence, the trend of cryo-EM structures having lower atomic packing scores than crystal structures is also observed at protein–protein interfaces. The difference in mean packing score values of cryo-EM and crystal structures at interfaces (0.046) is even larger than that seen in tertiary structures (0.017), suggesting that crystal packing forces potentially result in greater compaction of interfacial residues than present under native conditions.

**Figure 4:**
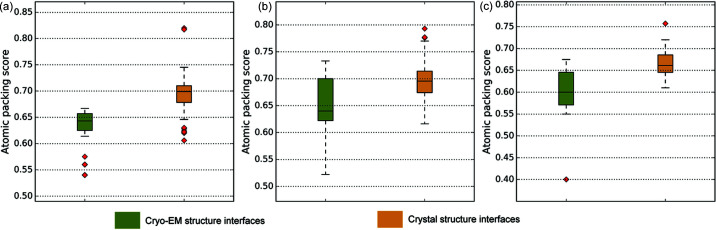
Comparison of interfacial packing scores between atomic models derived using cryo-EM and crystallography for the same proteins. Each subplot in the figure corresponds to 1 of 3 clusters of identical two-protein sub-assemblies in cryo-EM and crystal structures. The distribution of packing scores of interface residues in constituent cryo-EM (green) and crystal (yellow) structures is represented as boxplots in each subplot. (*a*) Interface of α and β subunits in 20S proteasome structures from Thermoplasma acidophilum. (*b*) Interface of α and β subunits in hemoglobin structures from humans. (*c*) Interface of mitchondrial cysteine desulfurase and mitochondrial iron–sulfur cluster assembly enzyme in humans.

In an extension of this analysis, we identified 16 clusters of chemically identical cryo-EM and crystal sub-assemblies such that, in each cluster, there is at least one sub-assembly structure determined using cryo-EM and a structure for the same sub-assembly was also determined using X-ray crystallography (Table S5). For the protein–protein complexes within each cluster, we compared the interface atomic packing scores of cryo-EM and crystal structures. We observe that the interfacial packing score in cryo-EM structures is lower than that seen in crystal structures in all clusters (Table S6). The difference is statistically significant in 12 out of the 16 clusters [*p* < 0.05; Mann–Whitney Test (exact values given in Table S6)]. For a more objective comparison, we selected a subset of these clusters that minimize the skew in number of interfaces between cryo-EM and crystal structures. The distribution of the packing score of interface residues for this subset of 3 clusters is shown in Fig. 4 ▸. Each panel in the figure shows the interfacial packing score distribution of a cluster. In all 3 clusters, the difference in interface packing score between cryo-EM and crystal structures is significant and consistent with the overall trend observed in non-interfacial regions.

## 4. Conclusions

We conclude that the inherent difference in the methods used for sample preparation in cryo-EM and X-ray crystallographic methods likely contributes to differences in packing scores between structures determined using these two methods. Factors such as lower water content and slower cooling rate of the crystals, which affect atomic positions of the protein structure, could lead to more compaction of side chains than that which occurs under native, fully hydrated conditions. Our analyses provide a quantitative measure of the extent of the difference in local compaction of protein side chains when structures are determined using cryo-EM or X-ray crystallography. Looser packing in cryo-EM structures is a reflection of higher interatomic spacing, which implies more room for movement of atoms without clashing with each other. We propose that this has implications in the following four aspects: determining the mechanism of action of proteins at active sites; studying the mechanistic features of function of proteins; modeling flexibility and dynamics of proteins; and modeling the extent of movement of atoms at drug binding sites. While performing these studies, one should give leeway for higher movement of atoms in crystal structures than what is deemed possible based on the static structure, because the structure of the same protein determined using cryo-EM would have higher interatomic spacing, which is also likely to be closer to the native state of the protein.

## Supplementary Material

Supporting figures and tables. DOI: 10.1107/S2052252521011945/fq5017sup1.pdf

## Figures and Tables

**Table 1:** Mean packing scores of cryo-EM and crystal (tertiary) structures in different resolution ranges

Resolution (in Å)	Crystal structures (*p* value)			Cryo-EM structures
	Set 1	Set 2	Set 3	
<2.5	0.730 (1.77×10^−3^)	0.730 (1.78×10^−3^)	0.731 (1.03×10^−3^)	0.722
≥2.5 and <3.0	0.725 (6.22×10^−5^)	0.727 (3.64×10^−5^)	0.725 (7.88×10^−5^)	0.714
≥3.0	0.721 (7.95×10^−5^)	0.721 (3.42×10^−5^)	0.727 (2.16×10^−8^)	0.711
