# Supplementary material for: Comparison of side-chain dispersion in protein structures determined by cryo-EM and X-ray crystallography
Source: IUCrJ. 2021 Dec 10;9(Pt 1):98–103. doi: 10.1107/S2052252521011945 (PMC8733892; doi:10.1107/S2052252521011945)
Supplement: Supplementary file 1 [file m-09-00098-sup1.pdf]

IUCrJ

Volume 9 (2022)

Supporting information for article:

Comparison of sidechain dispersion in protein structures determined by cryo-EM and X-ray crystallography

Ashraya Ravikumar, Mrugsen Nagsen Gopnarayan, Sriram Subramaniam and Narayanaswamy Srinivasan

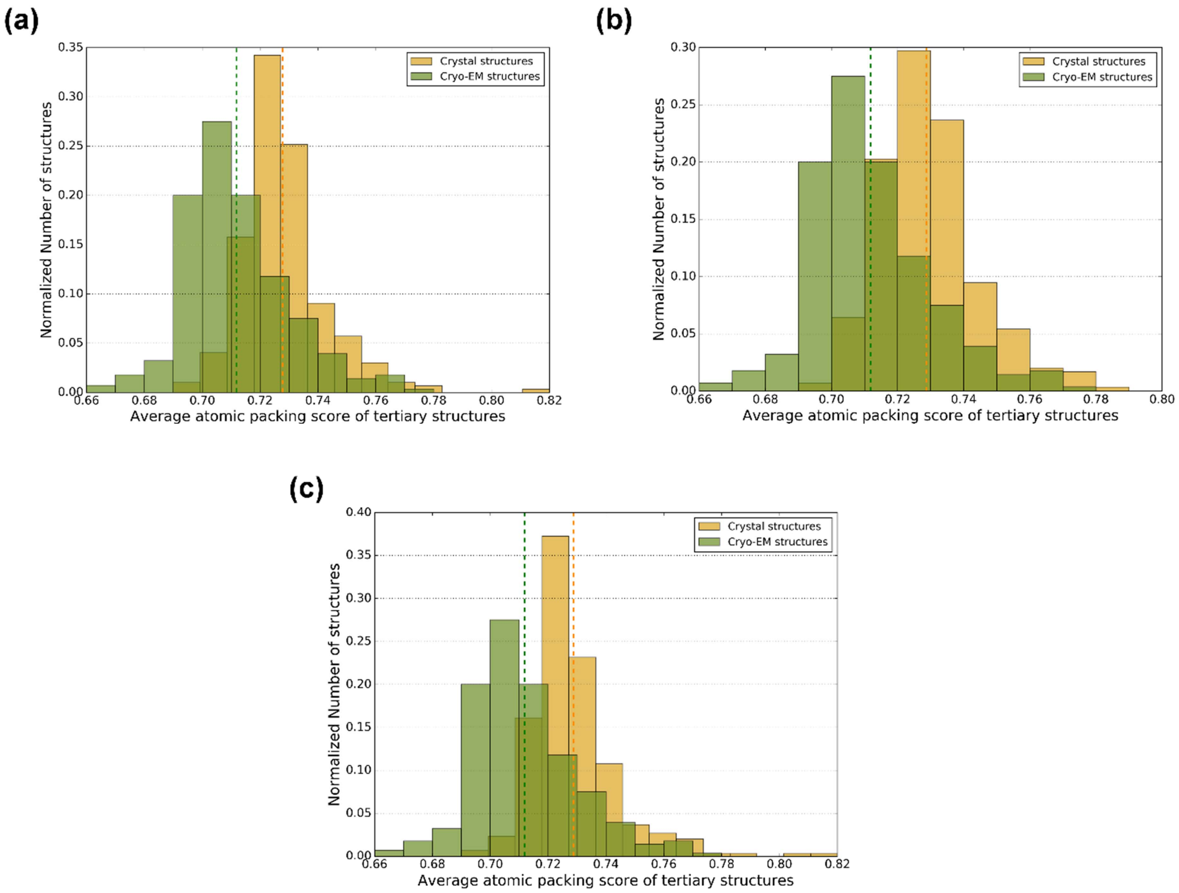

Supplementary Figure S1: Packing scores of tertiary structures of cryo-EM and crystal structures.

Average packing scores of cryo-EM structures and crystal structures from (a) Set 1, (b) Set 2 and (c) Set 3 where each set consists of 300 randomly chosen crystal structures

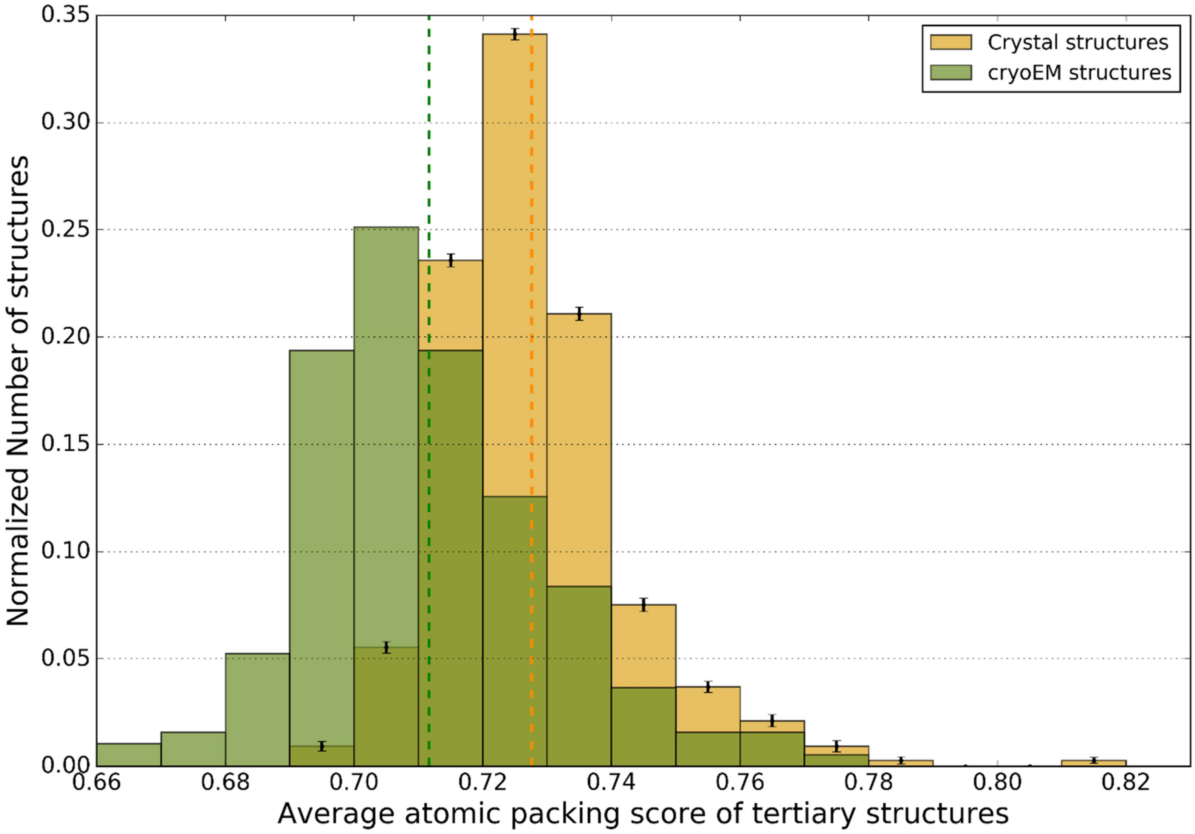

Supplementary Figure S2: Packing scores of tertiary structures of cryo-EM and crystal structures after filtering out residues in unusual environment recognized using WHATCHECK

This is similar to Figure 1, except that residues which have a Z-score < -5, as calculated by the coarse packing quality control module in WHATCHECK are not considered for the average packing score calculation of cryo-EM and crystal structures.

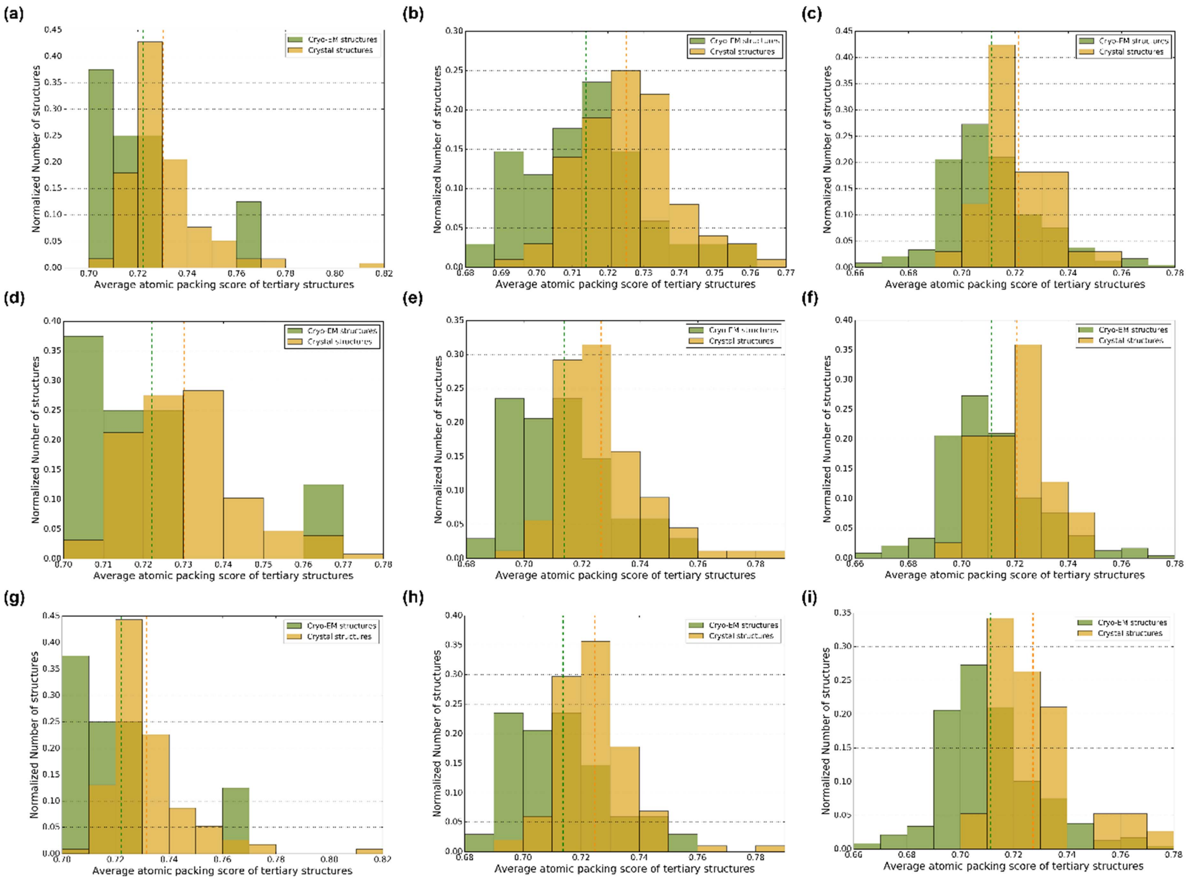

Supplementary Figure S3: Packing scores of tertiary structures of cryo-EM and crystal structures in similar resolution ranges

(a), (b) and (c) are comparisons of packing scores of crystal structures from Set 1 and cryo-EM structures and with resolution < 2.5Å, between 2.5 and 3Å and ≥ 3Å. Similarly, (d), (e) and (f) correspond to crystal structures from Set 2 and cryo-EM structures and (g), (h) and (i) correspond to crystal structures from Set 3 and cryo-EM structures. Mean values of the two distributions within each plot are shown as dashed lines. In all the plots, cryo-EM structures are shown in green and crystal structures are shown in yellow.

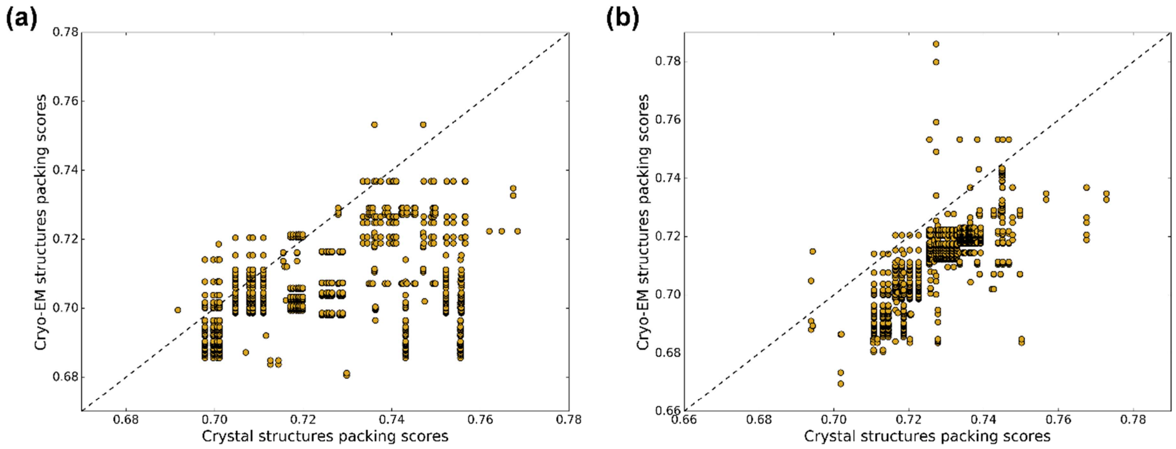

Supplementary Figure S4: Packing scores of pairs of same proteins solved by cryo-EM and X-ray crystallography

(a) Packing scores of crystal structures from Set 2 and cryo-EM structures of the same proteins. In 89% of the pairs, the crystal structure has a higher packing score than the cryo-EM structure, (b) Packing scores of crystal structures from Set 3 and cryo-EM structures of the same proteins. In 98% of the pairs, crystal structure has a higher packing score than the cryo-EM structure

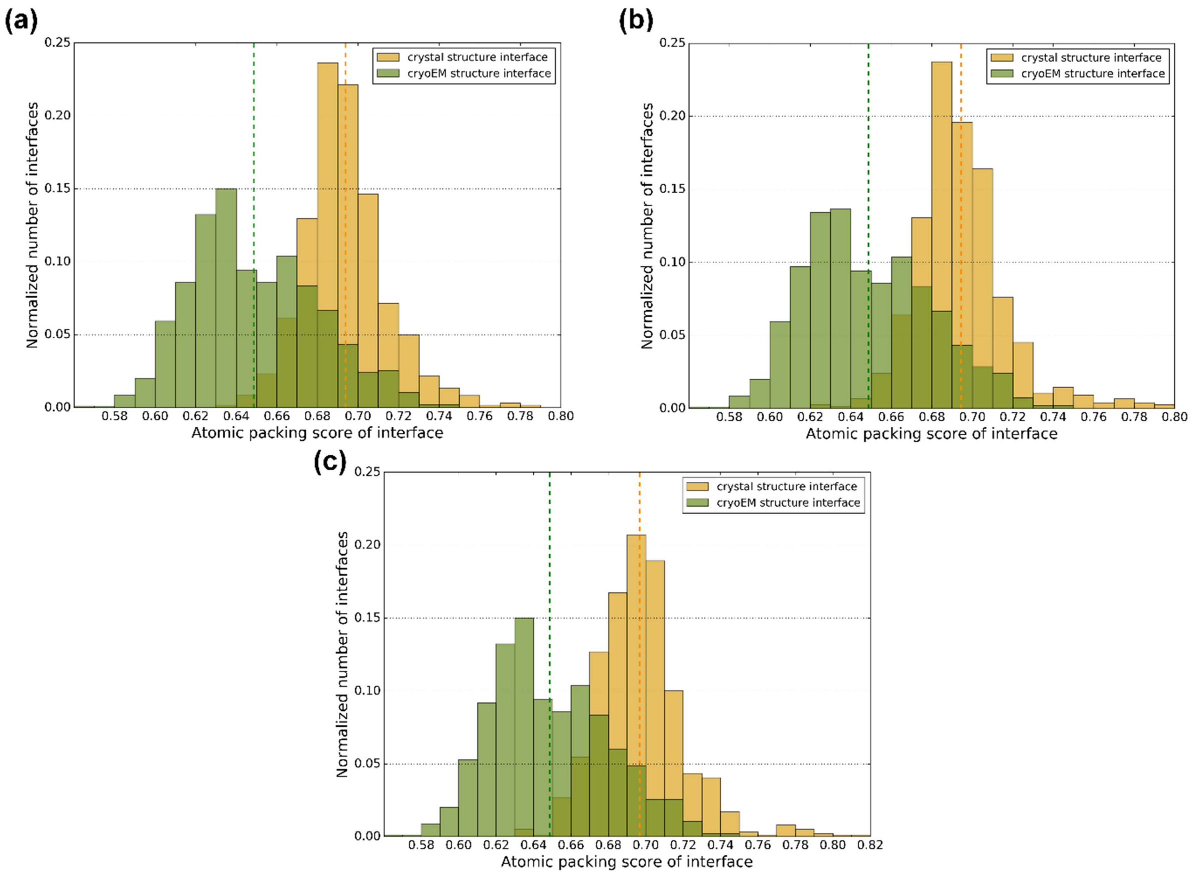

Supplementary Figure S5: Packing scores of interfaces between two protein sub-assemblies of cryo-EM structures and three sets of randomly chosen crystal structures

Packing scores of interfaces from cryo-EM structures and crystal structures from (a) Set 1, (b) Set 2 and (c) Set 3

Supplementary Table S1: List of cryo-EM structures' PDB codes used in this work

3j5p	5lzp	6a90	6csx	6fkf	6jcw	6nby	6rbk
3j7h	5n8n	6a91	6ct0	6fu8	6jcz	6nht	6rbn
3j9c	5ni1	6a95	6cud	6g2j	6jd1	6nhv	
3j9i	5nik	6acf	6cvj	6giq	6mb3	6niy	
3j9j	5nv3	6ach	6cvm	6gyb	6mdr	6njo	
3j9q	5oof	6adm	6dcq	6gyn	6mgv	6njp	
3jak	5syc	6adq	6dde	6gyo	6mgw	6nq1	
3jal	5sye	6agf	6ddf	6gyu	6mho	6nq2	
3jar	5syf	6ap1	6dg7	6h3i	6mhq	6nr3	
3jas	5syg	6aui	6djm	6h3n	6mhs	6nsj	
3jat	5t4d	6az0	6djn	6hbc	6mhv	6nsk	
3jbs	5tfy	6b3j	6dnf	6hbu	6mhy	6nt3	
3jck	5tj6	6baj	6dpu	6hcy	6mks	6nt4	
3jcu	5u6o	6bcj	6dpv	6hiq	6mlm	6nt9	
3jcz	5uj9	6bco	6dpw	6hjp	6mrt	6nyf	
3jd0	5uja	6bcq	6drj	6hls	6mru	6nyj	
3jd1	5vfo	6bdf	6drv	6hn5	6msm	6nyn	
3jd2	5vy3	6bgl	6dso	6hu9	6mwq	6nyy	
3jd4	5vy4	6bhu	6du8	6hug	6mzb	6nzu	
4ci0	5vy5	6bjc	6dw1	6huj	6mzu	6nzw	
4d3e	5w0s	6bly	6dwb	6hum	6mzv	6nzz	
4v1a	5w3e	6bmf	6e0g	6huo	6mzx	6o1n	
5a0q	5w3l	6bqr	6e1h	6hwh	6mzy	6o1p	
5a1a	5w3m	6bqv	6e1k	6hzm	6n06	6o1u	
5a63	5w3s	6btm	6e1m	6i1y	6n09	6o20	
5foj	5w5e	6c0v	6e3y	6i2k	6n1h	6o2r	
5ftj	5w5f	6c1d	6e7b	6i53	6n23	6o7t	
5ftk	5w68	6c24	6e7p	6idf	6n24	6o7u	
5ftl	5w81	6c26	6e9z	6igz	6n25	6o81	
5ftm	5wc3	6c6l	6ebk	6ihb	6n26	6o85	
5ftn	5wj9	6c70	6ebl	6ijj	6n27	6o9z	
5g05	5wq7	6c96	6et5	6ijo	6n28	6oeu	
5gaq	5wq8	6c9a	6eti	6ilk	6n2d	6oij	
5h1q	5xb1	6c9i	6eu2	6irs	6n2y	6on2	
5h3o	5xnl	6c9k	6eu3	6irt	6n2z	6qm7	
5i68	5xnm	6caj	6ezj	6iyc	6n30	6qm8	
5irx	5xno	6cjq	6ezm	6j0b	6n4b	6qnt	
5irz	5xtb	6cjt	6ezn	6j5t	6nb3	6qp6	
5is0	5xte	6cju	6f1t	6j8e	6nbb	6qpc	
5jxl	5yi5	6cmx	6f1u	6j8g	6nbc	6qpi	
5k0z	5z1w	6cnm	6f1v	6j8h	6nbd	6r3q	
5k12	5z96	6cnn	6f1y	6j8i	6nbf	6r4p	
5kmg	5zdh	6co7	6f1z	6j8j	6nbh	6r7x	
5lkh	5zji	6coy	6f95	6jb1	6nbq	6rao	
5lki	5zx5	6coz	6fhl	6jcv	6nbx	6rap	

Supplementary Table S2: List of crystal structures' PDB codes used in this work

Set 1

1ai4	1rhq	2qls	3k0c	4ewx	4zlk	5v8z	6pxg
1aif	1s1x	2qzx	3k16	4fnl	4zol	5vz5	6pzh
1ayb	1sc5	2r4r	3kka	4fu4	5aja	5wpa	6s8q
1bnd	1tii	2thf	3l4b	4g7y	5caw	5x8m	6sk3
1bvn	1tmf	2ux3	3ldx	4gyp	5cba	5xbp	6tz7
1bxr	1tmu	2v3b	3mrn	4hr7	5cfd	5xuq	6u2g
1cd1	1u91	2vdb	3mv3	4hrd	5cza	5yc2	6u9s
1d5s	1uea	2vvu	3oap	4imi	5czd	5yvw	6w0a
1dbb	1vrn	2w76	3oma	4j7c	5d1d	6a3e	6wiq
1dgr	1x76	2w99	3oxu	4jeh	5e3a	6az2	6xy4
1dm0	1xb1	2wa8	3qfb	4jff	5ejd	6b4j	6yuc
1dqm	1xvd	2wfl	3qnz	4jo1	5ez0	6bjp	6zcz
1dwe	1ya7	2wv4	3qxd	4jpz	5f96	6cp2	7joo
1dzi	1yie	2wyj	3rif	4jw3	5fir	6csu	
1e1r	1ykh	2x5u	3rk2	4k3p	5fo9	6dkp	
1eet	1ymm	2xa0	3rna	4lcx	5fwe	6ekm	
1ehk	1yth	2xc0	3sek	4lop	5gtb	6eqb	
1f4w	2adg	2ygg	3sf5	4m69	5gux	6et9	
1fg9	2adv	2yng	3tkl	4mg5	5hgv	6eyn	
1for	2arj	2zl2	3tl0	4n7z	5hi3	6fa2	
1frf	2b23	2zt9	3tnp	4nb8	5iq9	6fo8	
1gag	2b5u	3a5n	3u7d	4nfu	5isz	6fx4	
1gy3	2bap	3alo	3u88	4nja	5jy0	6gc2	
1him	2cpk	3aqf	3uc0	4nty	5kcu	6ghm	
1igf	2dd4	3at6	3vbf	4oud	5kji	6h7l	
1jbp	2ec6	3bky	3vbo	4pbz	5l5s	6hm8	
1jk0	2fnx	3bsz	3vu6	4rgm	5lhp	6htf	
1k5q	2fwo	3c2a	3zgx	4rhw	5lpn	6ian	
1kzo	2gac	3csn	3zms	4rw4	5lum	6j2h	
1mcc	2gvd	3d36	4a0i	4tpj	5lx9	6jcu	
1mcp	2h4m	3dpq	4a11	4tth	5nqk	6jez	
1mt8	2hnt	3ehb	4bbn	4tv8	5nw2	6ktm	
1nu7	2i0i	3eov	4bcm	4uad	5ogi	6lpr	
1nu9	2iy1	3f7d	4bfi	4uu9	5ovn	6mh2	
1o9f	2j6o	3fhc	4bvg	4xt1	5szj	6o5n	
1oay	2j7i	3gjn	4bx7	4xx0	5t0f	6o6e	
1oc9	2mha	3h3p	4c4v	4y5y	5t48	6p8f	
1prt	2ny7	3hym	4cdr	4y8v	5tml	6p8g	
1pst	2p9i	3i0r	4cvz	4ysy	5trp	6pfy	
1pyt	2pks	3it6	4dgc	4yxs	5tyi	6phh	
1qov	2pr9	3jvg	4e17	4z9v	5ufu	6plk	

Set 2

1a4j	1x2w	2x9a	3tn9	4nki	5i25	6b4r
1atp	1xx9	2xa7	3tuf	4om0	5icz	6b5l
1buv	1xxf	2zme	3tuz	4org	5ii0	6cga
1cfv	1y01	2zxx	3tyf	4p4h	5j9q	6co1
1cwb	1y31	3bhb	3u1s	4pln	5jo1	6cpm
1d3q	1y5j	3bqd	3uio	4qic	5k20	6d68
1e1c	1ya7	3c98	3v4o	4qlu	5l6h	6dmx
1e1r	1yrq	3du2	3wfd	4r9u	5las	6e9p
1fev	1zc4	3dvn	3wr1	4rw7	5lp6	6fmq
1fk9	1zh7	3eoy	3zpv	4rw9	5lxg	6gc2
1gbi	1zt4	3fb6	4bea	4u1l	5ma6	6gkg
1gec	2a4q	3fo2	4bj5	4udu	5mb9	6h2a
1gzp	2a4r	3g5z	4bv0	4uhy	5nw8	6hk4
1h2l	2a5y	3gfk	4bxf	4uua	5om8	6htr
1hdb	2afh	3gjo	4c11	4v0c	5omc	6hw6
1iqm	2an6	3gjq	4c2f	4wkm	5or2	6ieb
1izn	2arj	3h1h	4cdw	4x8w	5q0f	6j4o
1jh0	2b2w	3h5c	4chg	4xkl	5q0y	6jep
1jql	2be7	3h9q	4cni	4xyi	5qtx	6jf6
1ktk	2bha	3i7o	4cqv	4yf9	5t3r	6ks1
1kzo	2cly	3ifw	4czd	4yk4	5tlg	6m93
1ljw	2cvy	3ijh	4eb6	4z0b	5tp1	6myp
1lmw	2d1j	3jvg	4f9l	4zny	5tsv	6nbl
1m71	2dvq	3kee	4fa1	4zxd	5tzt	6nux
1mkx	2dxb	3kka	4fap	5aaw	5u1r	6nwr
1nek	2e3k	3l4f	4ffv	5bwe	5u1t	6oan
1nih	2efd	3mck	4g2v	5cw7	5u68	6ob3
1oqn	2ey4	3mqp	4hr6	5cww	5va6	6okm
1oy3	2fo5	3na1	4hs3	5d1x	5vbc	6pa0
1p9u	2hh0	3nth	4hwi	5dke	5vie	6phf
1pek	2ht3	3o2m	4ia7	5dxp	5vz4	6qb4
1pg5	2ih1	3oap	4ifk	5ejv	5wgb	6qrm
1pss	2iou	3or0	4ify	5eya	5xkj	6qv0
1qsc	2j4w	3our	4inr	5fer	5xuq	6rr0
1rv6	2mha	3oy6	4jas	5fge	5xzx	6ue5
1s7w	2ot3	3pxs	4jol	5fp2	5ylv	6uig
1tdq	2uum	3pxt	4jzn	5ggu	5ymv	6v0n
1to9	2v8y	3qhe	4k0u	5ghs	5yy5	6vzv
1tsu	2vbc	3qq3	4k8r	5gre	5yz3	6wt3
1umc	2vpw	3qt2	4l2k	5gsd	5zpw	6x5e
1urc	2w2q	3rev	4mjs	5h7y	5ztb	7ci2
1vbb	2wxz	3sf5	4mms	5hho	5zut	7jur
1wu1	2x4r	3t6b	4n7n	5hpy	6b4h	

Set 3

1acy	1svh	2y9m	3nqj	4nbe	5i70	6bd2
1ao7	1t21	2yjn	3ogl	4ni0	5idn	6biv
1b34	1tny	2yxq	3p5t	4niq	5if1	6d0k
1bcp	1twg	2zgh	3pqy	4nnp	5jkr	6d43
1bwu	1x75	2zxw	3qh3	4nnw	5jor	6dcn
1bzh	1xtc	3a5o	3qib	4no1	5kkz	6dlb
1cmi	1xvd	3aso	3rhw	4np4	5koa	6dm9
1cu4	1yn7	3bdm	3rlm	4nwq	5l5a	6dy0
1ds5	1zky	3c14	3stz	4o4i	5m5u	6et2
1ezx	2b4s	3c5w	3uqr	4olm	5men	6ex9
1ezz	2b5u	3ch1	3uvl	4or9	5mlw	6fbj
1f5q	2b7b	3d1a	3vu5	4p5t	5moy	6huc
1fj1	2ba1	3dbl	3w2v	4pxh	5n52	6i07
1g2c	2be9	3dvu	3zc7	4q4i	5nsp	6icx
1h1p	2bp7	3e0m	3zil	4q4j	5nwj	6j2j
1h2u	2bq7	3e4z	4auq	4qo2	5opy	6jlk
1hba	2ccl	3eh3	4bwg	4qok	5ovw	6jpr
1huc	2co2	3eh5	4bxe	4quf	5tm6	6k4r
1ii4	2f9d	3epa	4c0d	4qv6	5u1r	6kwk
1jbp	2flk	3eyo	4caa	4qvv	5uiw	6lkv
1jvz	2gsi	3fyu	4d0b	4u6v	5un7	6ln2
1kcg	2hbd	3gjr	4djz	4u8u	5uwo	6lvv
1kj3	2hh0	3hd7	4edn	4uec	5uwt	6obp
1kkl	2hpq	3hdi	4ers	4uqi	5v4n	6p02
1kyi	2i5y	3hqh	4ftv	4uub	5vjl	6p9j
1kzg	2ibi	3i4d	4gb3	4wwi	5vnj	6phf
1l0a	2iw6	3ig1	4gto	4x8w	5wak	6s24
1lb5	2j95	3ijh	4gtr	4y7o	5wgg	6seh
1lw0	2jh5	3jsv	4h1w	4ybq	5wlw	6sxn
1m72	2occ	3k9p	4hb3	4yeb	5wna	6tcn
1mci	2oxh	3kbt	4hkz	4yn0	5wsv	6tej
1mda	2p28	3kj6	4hrh	4ywc	5x3o	6u36
1nfw	2qt5	3kka	4i7d	5bnw	5x3x	6ugt
1ngy	2siv	3kl4	4ij3	5c2t	5xxy	6utu
1ni1	2trs	3koz	4isq	5c6p	5yvh	6vhr
1nm5	2uud	3krd	4j30	5cz9	5ywf	6w0h
1nsn	2uul	3kyc	4jo4	5d1q	5zk5	6wuu
1pg5	2v3b	3l0e	4joi	5de2	5zwi	6x49
1qo0	2vas	3l9i	4jra	5di7	6ajl	6y6d
1rhj	2vxu	3lqd	4lte	5dob	6al4	6yjp
1rhm	2ws3	3mgt	4lwy	5dxb	6at1	6z1n
1sbb	2wv5	3mro	4m48	5fu7	6b2a	7jx3
1sky	2x1n	3my5	4n7k	5hyn	6bc0	

Supplementary Table S3: Mean packing scores of cryo-EM and crystal (tertiary) structures in fine resolution bin widths

Resolution (in Å)	Average packing score of cryo-EM structures	Average packing score of crystal structures	Number of cryo-EM structures	Number of crystal structures
<2.2	0.72803497	0.73480688	3	36
2.2 to 2.4	0.71596147	0.73093072	4	58
2.4 to 2.6	0.7208233	0.72736199	2	37
2.6 to 2.8	0.70905761	0.72311286	8	50
2.8 to 3.0	0.71529669	0.72552738	25	35
3.0 to 3.2	0.70318375	0.72643051	50	19
3.2 to 3.4	0.71210795	0.72480286	98	14
>=3.4	0.71304574	0.73171162	90	5

Supplementary Table S4: Comparison of mean packing scores of pairs of same proteins solved by cryo-EM and X-ray crystallography before and after removing pairs of structures with resolution difference > 0.5Å

	% of crystal structures with higher packing score than cryo-EM counterparts	
	Before accounting for resolution difference	After accounting for resolution difference
Set 1 crystal vs. cryo-EM structures	92	96
Set 2 crystal vs. cryo-EM structures	89	85
Set 3 crystal vs. cryo-EM structures	98	92

Supplementary Table S5: List of sequentially identical two-protein sub-assemblies in cryo-EM and crystal structures

Identical sequence cluster number	PDB code_chain1_chain2	Category
1	5ni1_A_B	cryo-EM structure interface
1	5ni1_C_D	cryo-EM structure interface
1	6nbc_A_B	cryo-EM structure interface
1	6nbc_C_D	cryo-EM structure interface
1	6nbd_A_B	cryo-EM structure interface
1	6nbd_C_D	cryo-EM structure interface
1	1yie_A_B	crystal structure interface
1	1yie_C_D	crystal structure interface
2	3jak_A_B	cryo-EM structure interface
2	3jak_B_K	cryo-EM structure interface
2	3jak_C_D	cryo-EM structure interface
2	3jak_D_L	cryo-EM structure interface
2	3jak_E_F	cryo-EM structure interface
2	3jak_F_J	cryo-EM structure interface
2	3jak_E_G	cryo-EM structure interface
2	3jak_A_H	cryo-EM structure interface
2	3jak_C_I	cryo-EM structure interface
2	3jal_A_B	cryo-EM structure interface
2	3jal_B_K	cryo-EM structure interface
2	3jal_C_D	cryo-EM structure interface
2	3jal_D_L	cryo-EM structure interface
2	3jal_E_F	cryo-EM structure interface
2	3jal_F_J	cryo-EM structure interface
2	3jal_E_G	cryo-EM structure interface
2	3jal_A_H	cryo-EM structure interface
2	3jal_C_I	cryo-EM structure interface
2	3jar_A_B	cryo-EM structure interface
2	3jar_B_K	cryo-EM structure interface
2	3jar_C_D	cryo-EM structure interface
2	3jar_D_L	cryo-EM structure interface
2	3jar_E_F	cryo-EM structure interface
2	3jar_F_J	cryo-EM structure interface
2	3jar_E_G	cryo-EM structure interface
2	3jar_A_H	cryo-EM structure interface
2	3jar_C_I	cryo-EM structure interface
2	3jas_A_B	cryo-EM structure interface
2	3jas_B_K	cryo-EM structure interface
2	3jas_C_D	cryo-EM structure interface
2	3jas_D_L	cryo-EM structure interface
2	3jas_E_F	cryo-EM structure interface

2	3jas_F_J	cryo-EM structure interface
2	3jas_E_G	cryo-EM structure interface
2	3jas_A_H	cryo-EM structure interface
2	3jas_C_I	cryo-EM structure interface
2	3jat_A_B	cryo-EM structure interface
2	3jat_B_K	cryo-EM structure interface
2	3jat_C_D	cryo-EM structure interface
2	3jat_D_L	cryo-EM structure interface
2	3jat_E_F	cryo-EM structure interface
2	3jat_F_J	cryo-EM structure interface
2	3jat_E_G	cryo-EM structure interface
2	3jat_A_H	cryo-EM structure interface
2	3jat_C_I	cryo-EM structure interface
2	5kmg_A_B	cryo-EM structure interface
2	6bjc_A_B	cryo-EM structure interface
2	6bjc_B_K	cryo-EM structure interface
2	6bjc_C_D	cryo-EM structure interface
2	6bjc_D_L	cryo-EM structure interface
2	6bjc_E_F	cryo-EM structure interface
2	6bjc_F_J	cryo-EM structure interface
2	6bjc_E_G	cryo-EM structure interface
2	6bjc_A_H	cryo-EM structure interface
2	6bjc_C_I	cryo-EM structure interface
2	6cvj_A_B	cryo-EM structure interface
2	6cvj_A_C	cryo-EM structure interface
2	6dpu_A_B	cryo-EM structure interface
2	6dpu_B_K	cryo-EM structure interface
2	6dpu_C_D	cryo-EM structure interface
2	6dpu_D_L	cryo-EM structure interface
2	6dpu_E_F	cryo-EM structure interface
2	6dpu_F_J	cryo-EM structure interface
2	6dpu_E_G	cryo-EM structure interface
2	6dpu_A_H	cryo-EM structure interface
2	6dpu_C_I	cryo-EM structure interface
2	6dpv_A_B	cryo-EM structure interface
2	6dpv_B_K	cryo-EM structure interface
2	6dpv_C_D	cryo-EM structure interface
2	6dpv_D_L	cryo-EM structure interface
2	6dpv_E_F	cryo-EM structure interface
2	6dpv_F_J	cryo-EM structure interface
2	6dpv_E_G	cryo-EM structure interface
2	6dpv_A_H	cryo-EM structure interface
2	6dpv_C_I	cryo-EM structure interface
2	6dpw_A_B	cryo-EM structure interface
2	6dpw_B_K	cryo-EM structure interface
2	6dpw_C_D	cryo-EM structure interface
2	6dpw_D_L	cryo-EM structure interface
2	6dpw_E_F	cryo-EM structure interface

2	6dpw_F_J	cryo-EM structure interface
2	6dpw_E_G	cryo-EM structure interface
2	6dpw_A_H	cryo-EM structure interface
2	6o2r_A_B	cryo-EM structure interface
2	6o2r_B_K	cryo-EM structure interface
2	6o2r_C_D	cryo-EM structure interface
2	6o2r_D_L	cryo-EM structure interface
2	6o2r_E_F	cryo-EM structure interface
2	6o2r_F_J	cryo-EM structure interface
2	6o2r_A_H	cryo-EM structure interface
2	6o2r_C_I	cryo-EM structure interface
2	4eb6_A_B	crystal structure interface
2	3jak_A_B	cryo-EM structure interface
2	3jak_A_H	cryo-EM structure interface
2	3jak_C_D	cryo-EM structure interface
2	3jak_C_I	cryo-EM structure interface
2	3jak_E_F	cryo-EM structure interface
2	3jak_E_G	cryo-EM structure interface
2	3jak_F_J	cryo-EM structure interface
2	3jak_B_K	cryo-EM structure interface
2	3jak_D_L	cryo-EM structure interface
2	3jal_A_B	cryo-EM structure interface
2	3jal_A_H	cryo-EM structure interface
2	3jal_C_D	cryo-EM structure interface
2	3jal_C_I	cryo-EM structure interface
2	3jal_E_F	cryo-EM structure interface
2	3jal_E_G	cryo-EM structure interface
2	3jal_F_J	cryo-EM structure interface
2	3jal_B_K	cryo-EM structure interface
2	3jal_D_L	cryo-EM structure interface
2	3jar_A_B	cryo-EM structure interface
2	3jar_A_H	cryo-EM structure interface
2	3jar_C_D	cryo-EM structure interface
2	3jar_C_I	cryo-EM structure interface
2	3jar_E_F	cryo-EM structure interface
2	3jar_E_G	cryo-EM structure interface
2	3jar_F_J	cryo-EM structure interface
2	3jar_B_K	cryo-EM structure interface
2	3jar_D_L	cryo-EM structure interface
2	3jas_A_B	cryo-EM structure interface
2	3jas_A_H	cryo-EM structure interface
2	3jas_C_D	cryo-EM structure interface
2	3jas_C_I	cryo-EM structure interface
2	3jas_E_F	cryo-EM structure interface
2	3jas_E_G	cryo-EM structure interface
2	3jas_F_J	cryo-EM structure interface
2	3jas_B_K	cryo-EM structure interface
2	3jas_D_L	cryo-EM structure interface

2	3jat_A_B	cryo-EM structure interface
2	3jat_A_H	cryo-EM structure interface
2	3jat_C_D	cryo-EM structure interface
2	3jat_C_I	cryo-EM structure interface
2	3jat_E_F	cryo-EM structure interface
2	3jat_E_G	cryo-EM structure interface
2	3jat_F_J	cryo-EM structure interface
2	3jat_B_K	cryo-EM structure interface
2	3jat_D_L	cryo-EM structure interface
2	5kmg_A_B	cryo-EM structure interface
2	6bjc_A_B	cryo-EM structure interface
2	6bjc_A_H	cryo-EM structure interface
2	6bjc_C_D	cryo-EM structure interface
2	6bjc_C_I	cryo-EM structure interface
2	6bjc_E_F	cryo-EM structure interface
2	6bjc_E_G	cryo-EM structure interface
2	6bjc_F_J	cryo-EM structure interface
2	6bjc_B_K	cryo-EM structure interface
2	6bjc_D_L	cryo-EM structure interface
2	6cvj_A_B	cryo-EM structure interface
2	6cvj_A_C	cryo-EM structure interface
2	6dpu_A_B	cryo-EM structure interface
2	6dpu_A_H	cryo-EM structure interface
2	6dpu_C_D	cryo-EM structure interface
2	6dpu_C_I	cryo-EM structure interface
2	6dpu_E_F	cryo-EM structure interface
2	6dpu_E_G	cryo-EM structure interface
2	6dpu_F_J	cryo-EM structure interface
2	6dpu_B_K	cryo-EM structure interface
2	6dpu_D_L	cryo-EM structure interface
2	6dpv_A_B	cryo-EM structure interface
2	6dpv_A_H	cryo-EM structure interface
2	6dpv_C_D	cryo-EM structure interface
2	6dpv_C_I	cryo-EM structure interface
2	6dpv_E_F	cryo-EM structure interface
2	6dpv_E_G	cryo-EM structure interface
2	6dpv_F_J	cryo-EM structure interface
2	6dpv_B_K	cryo-EM structure interface
2	6dpv_D_L	cryo-EM structure interface
2	6dpw_A_B	cryo-EM structure interface
2	6dpw_A_H	cryo-EM structure interface
2	6dpw_C_D	cryo-EM structure interface
2	6dpw_E_F	cryo-EM structure interface
2	6dpw_E_G	cryo-EM structure interface
2	6dpw_F_J	cryo-EM structure interface
2	6dpw_B_K	cryo-EM structure interface
2	6dpw_D_L	cryo-EM structure interface
2	6o2r_A_B	cryo-EM structure interface

2	6o2r_A_H	cryo-EM structure interface
2	6o2r_C_D	cryo-EM structure interface
2	6o2r_C_I	cryo-EM structure interface
2	6o2r_E_F	cryo-EM structure interface
2	6o2r_F_J	cryo-EM structure interface
2	6o2r_B_K	cryo-EM structure interface
2	6o2r_D_L	cryo-EM structure interface
2	4eb6_B_C	crystal structure interface
2	4eb6_C_D	crystal structure interface
2	5lp6_A_B	crystal structure interface
2	5lp6_B_C	crystal structure interface
2	5lp6_C_D	crystal structure interface
2	5yz3_A_B	crystal structure interface
2	5yz3_B_C	crystal structure interface
2	5yz3_C_D	crystal structure interface
3	6et5_C_M	cryo-EM structure interface
3	1vrn_C_M	crystal structure interface
3	2x5u_C_M	crystal structure interface
4	6et5_C_L	cryo-EM structure interface
4	1vrn_C_L	crystal structure interface
4	2x5u_C_L	crystal structure interface
5	6et5_L_M	cryo-EM structure interface
5	1vrn_L_M	crystal structure interface
5	2x5u_L_M	crystal structure interface
6	6et5_H_M	cryo-EM structure interface
6	1vrn_H_M	crystal structure interface
6	2x5u_H_M	crystal structure interface
7	6et5_H_L	cryo-EM structure interface
7	1vrn_H_L	crystal structure interface
7	2x5u_H_L	crystal structure interface
8	3j9i_1_S	cryo-EM structure interface
8	3j9i_2_T	cryo-EM structure interface
8	3j9i_G_H	cryo-EM structure interface
8	3j9i_A_I	cryo-EM structure interface
8	3j9i_B_J	cryo-EM structure interface
8	3j9i_C_K	cryo-EM structure interface
8	3j9i_D_L	cryo-EM structure interface
8	3j9i_E_M	cryo-EM structure interface
8	3j9i_F_N	cryo-EM structure interface
8	3j9i_U_V	cryo-EM structure interface
8	3j9i_O_W	cryo-EM structure interface
8	3j9i_P_X	cryo-EM structure interface
8	3j9i_Q_Y	cryo-EM structure interface
8	3j9i_R_Z	cryo-EM structure interface
8	6bdf_1_Y	cryo-EM structure interface
8	6bdf_B_C	cryo-EM structure interface
8	6bdf_D_E	cryo-EM structure interface
8	6bdf_F_G	cryo-EM structure interface

8	6bdf_H_I	cryo-EM structure interface
8	6bdf_J_K	cryo-EM structure interface
8	6bdf_L_M	cryo-EM structure interface
8	6bdf_A_N	cryo-EM structure interface
8	6bdf_0_P	cryo-EM structure interface
8	6bdf_O_R	cryo-EM structure interface
8	6bdf_Q_T	cryo-EM structure interface
8	6bdf_S_V	cryo-EM structure interface
8	6bdf_U_X	cryo-EM structure interface
8	6bdf_W_Z	cryo-EM structure interface
8	1ya7_A_H	crystal structure interface
8	1ya7_B_I	crystal structure interface
8	1ya7_C_J	crystal structure interface
8	1ya7_D_K	crystal structure interface
8	1ya7_E_L	crystal structure interface
8	1ya7_F_M	crystal structure interface
8	1ya7_G_N	crystal structure interface
9	6i2k_B_C	cryo-EM structure interface
9	3vbf_B_C	crystal structure interface
9	3vbo_B_C	crystal structure interface
10	6i2k_A_B	cryo-EM structure interface
10	4cdw_A_B	crystal structure interface
11	6i2k_A_C	cryo-EM structure interface
11	4cdw_A_C	crystal structure interface
12	6c24_K_M	cryo-EM structure interface
12	5hyn_A_C	crystal structure interface
12	5hyn_F_H	crystal structure interface
12	5hyn_K_M	crystal structure interface
12	5hyn_Q_S	crystal structure interface
13	6c24_N_Q	cryo-EM structure interface
13	5wak_A_B	crystal structure interface
14	6c24_C_L	cryo-EM structure interface
14	5hyn_A_B	crystal structure interface
14	5hyn_F_G	crystal structure interface
14	5hyn_K_L	crystal structure interface
14	5hyn_Q_R	crystal structure interface
15	6nzu_A_D	cryo-EM structure interface
15	6nzu_E_H	cryo-EM structure interface
15	5wlw_A_D	crystal structure interface
15	5wlw_E_H	crystal structure interface
16	5ni1_A_B	cryo-EM structure interface
16	5ni1_C_D	cryo-EM structure interface
16	6nbc_A_B	cryo-EM structure interface
16	6nbc_C_D	cryo-EM structure interface
16	6nbd_A_B	cryo-EM structure interface
16	6nbd_C_D	cryo-EM structure interface
16	1hba_A_B	crystal structure interface
16	5ni1_A_B	cryo-EM structure interface

16	5ni1_C_D	cryo-EM structure interface
16	6nbc_A_B	cryo-EM structure interface
16	6nbc_C_D	cryo-EM structure interface
16	6nbd_A_B	cryo-EM structure interface
16	6nbd_C_D	cryo-EM structure interface
16	1hba_B_C	crystal structure interface
16	1hba_C_D	crystal structure interface
16	2hbd_A_B	crystal structure interface
16	4ni0_A_B	crystal structure interface

Table S6: Packing at protein-protein interfaces of cryo-EM and crystal structures of clusters of same two-protein sub-assembles

S.No	Packing at cryo-EM protein-protein interfaces	Packing at crystal protein-protein interfaces	p-value
1	0.655	0.7	1.20×10^{-3}
2	0.644	0.722	$<10^{-16}$
3	0.633	0.684	3.21×10^{-5}
4	0.651	0.671	2.21×10^{-1}
5	0.654	0.698	2.97×10^{-11}
6	0.631	0.689	1.02×10^{-5}
7	0.685	0.666	2.80×10^{-1}
8	0.631	0.693	$<10^{-16}$
9	0.605	0.677	4.34×10^{-12}
10	0.62	0.684	1.12×10^{-10}
11	0.623	0.681	1.15×10^{-09}
12	0.68	0.694	6.9×10^{-2}
13	0.632	0.696	1.39×10^{-4}
14	0.66	0.672	3.04×10^{-1}
15	0.601	0.67	3.44×10^{-5}
16	0.655	0.691	5.31×10^{-5}
